# Clinical Outcomes of Maxillary Sinus Floor Perforation by Dental Implants and Sinus Membrane Perforation during Sinus Augmentation: A Systematic Review and Meta-Analysis

**DOI:** 10.3390/jcm13051253

**Published:** 2024-02-22

**Authors:** Yousef Mohamed Sala, Hans Lu, Bruno Ramos Chrcanovic

**Affiliations:** 1 Faculty of Odontology, Malmö University, SE-214 21 Malmö, Sweden; jasufesaleh1@hotmail.com (Y.M.S.); hans_1997@live.se (H.L.); 2 Department of Oral and Maxillofacial Surgery and Oral Medicine, Faculty of Odontology, Malmö University, SE-214 21 Malmö, Sweden

**Keywords:** dental implant, maxillary sinus, sinus membrane, perforation, failure, systematic review, meta-analysis

## Abstract

The aim of the present systematic review was to investigate the clinical outcomes after the perforation of the maxillary sinus by dental implants, or after maxillary sinus membrane perforation during sinus lift procedure. Twenty-nine publications were included. Failure rates of implants in cases where perforation of sinus floor had happened (11 studies) was generally low, and only one case of transient sinusitis was reported. The estimated failure rate of these implants was 2.1% (SE 1.0%, *p* = 0.035). There were 1817 implants (73 failures) placed in augmented sinuses in which the sinus membrane was perforated and 5043 implants (274 failures) placed in sinuses with no perforated membrane, from 18 studies. The odds of implant failure difference between the groups were not significant (OR 1.347, *p* = 0.197). log OR of implant failure between perforated and non-perforated membrane groups did not significantly change with the follow-up time (−0.004/month; *p* = 0.500). In conclusion, implant failure rate is generally low either for implants penetrating in the floor of the maxillary sinus or implants placed in augmented sinuses in which the sinus membrane was perforated. The prevalence of postoperative infection/sinusitis is low, and it may depend either on the dimensions of the perforation or on the anatomical predisposition.

## 1. Introduction

The area of the posterior maxilla is recognized by the presence of the maxillary sinuses, bilateral cavities that usually compromises the volume of bone available in the region [1]. The local shortage of bone is aggravated by pneumatization, the progressive expansion of the sinuses volume, a process that begins after birth and continues until the 2nd–3rd decades of life [2]. The loss of posterior maxillary teeth exacerbates the situation, due to additional pneumatization [3], and resorption of the alveolar bone, resulting in loss of bone height [4]. Moreover, the region is typically characterized by trabecular bone of low density and thin cortical bone, which may exert a negative impact on implant survival [5]. Some options for the rehabilitation of the posterior maxilla, when this region becomes edentulous due to loss of teeth, include the placement of implants anchored in the pterygoid process [6] or in the zygomatic bone [7], each approach with their advantages and risks, with potential complications more commonly observed with zygomatic implants [8,9,10]. Two other options consist of the installation of short dental implants, and grafting of the maxillary sinus floor.

Implants of short lengths can be placed when the bone is low in height. There is, however, a recommendation that the implant should be anchored to a minimum of 4 mm of bone to increase the possibility of primary stability, and consequently reduce the risk of early implant loss [11]. This is not always possible though, which prompts the health professional to consider bone grafting.

The approach of grafting the maxillary sinus is called of maxillary sinus elevation surgery, or maxillary sinus lift/augmentation. In this technique, graft material is added to the floor of the sinus to increase the height of available bone, so implants of longer lengths can be placed [12]. Complications can, however, occur, such as tearing of the (Schneiderian) sinus membrane, bleeding, infection, loss of the graft material, persistent sinusitis, among others [13].

Perforation of the sinus membrane can happen or not after perforation of the maxillary sinus floor when a dental implant is placed without sinus augmentation. No adverse effects may result when the penetration of the implant into the sinus is small, namely, less than 2 mm, and the membrane is not perforated. However, when the implant intrusion into the sinus is greater and the membrane is perforated, complications like epistaxis, thickening of the membrane, and sinusitis can occur [14].

The purpose of the present study was to investigate the implant failure rates and complications after the perforation of the maxillary sinus floor by the placement of dental implants in posterior region of the maxilla, or after maxillary sinus membrane perforation during sinus lift procedure, based on a systematic review of the literature.

## 2. Materials and Methods

This study followed the PRISMA 2020 Statement guidelines [15]. Register in PROSPERO was undertaken with the registration number CRD42024497046.

### 2.1. Focused Question

The focused question was elaborated using the participants, interventions, comparisons, outcomes (PICO) format: In patients being rehabilitated with dental implants, what is the implant failure rates and complications after either maxillary sinus perforation due to the placement of dental implants in the posterior maxilla, or maxillary sinus membrane perforation during sinus lift procedure in comparison to non-perforated cases?

### 2.2. Search Strategies

An electronic search without time restrictions was first undertaken in September 2022, and the last update occurred in October 2023, in the following databases: PubMed/Medline, Web of Science, and Science Direct. The following terms were used in the search strategies:

(“dental implant” OR “oral implant”) AND (“maxillary sinus” OR “maxillary sinus membrane” OR “Schneiderian membrane”) AND (perforation OR perforate OR penetration OR intrusion).

A manual search of dental implants-related journals was performed (the list of journals can be found in the Supplementary Materials). The reference lists of the identified studies and the relevant reviews on the subject were also checked for possible additional studies.

### 2.3. Inclusion and Exclusion Criteria

Eligibility criteria included clinical human studies providing information on patients rehabilitated with dental implants in the posterior maxilla which reported either perforation of the maxillary sinus, or perforation of the maxillary sinus membrane during the procedure of sinus lift. Only the cases rehabilitated with cylindrical screw-type modern dental implants of titanium (commercially pure Ti) or its alloys were considered.

Exclusion criteria were case reports, technical reports, animal studies, in vitro studies, and reviews papers. It was also excluded studies reporting cases rehabilitated with mini-implants, zygomatic, orthodontic, zirconia, subperiosteal, or hollow implants.

### 2.4. Study Selection

The titles and abstracts of all reports identified through the electronic searches were read independently by the three authors. For studies appearing to meet the inclusion criteria, or for which there were insufficient data in the title and abstract to make a clear decision, the full report was obtained. Disagreements were solved by discussion between the authors.

### 2.5. Quality Assessment

Quality assessment of the studies was executed according to the Quality Assessment Tool of the National Institutes of Health [16]. Studies of “good” quality were judged to have at least 7 points.

### 2.6. Definitions

Sinus perforation: Implant perforation of the maxillary sinus was defined as the intrusion of a dental implant into the sinus cavity during drilling or implant placement, with and without punch out Schneiderian membrane [14].

Sinus lift: Sinus lift procedures increase bone volume by augmenting the sinus cavity with autogenous bone and/or commercially available biomaterials [17].

Implant failure: An implant was considered a failure if presenting signs and symptoms that led to implant removal, i.e., a lost implant. Implant failure could be either early (the inadequacy of the host to establish or promote osseointegration in the early stages of healing) or late (the failure of either the established osseointegration or function of dental implants) [18]. Fracture of an implant was also considered as a failure [19].

Complications consisted of clinical or radiographic complications. Clinical complications: sinusitis, nasal bleeding, nasal obstruction, nasal secretion, headache, pain/tenderness in the sinus region, decreased sense of smell. Radiographic complications: thickening of Schneiderian membrane, bone reaction to the implants, sinus pathology.

### 2.7. Data Extraction

The following data were extracted: year of publication, study design, country, study setting, number of patients, patients’ age and sex, implant healing period, failed and placed implants with sinus perforation or with sinus membrane perforation during sinus augmentation, implant system, presence of smokers in the patients’ study group, occurrence of complications, and follow-up time. Contact with authors for providing missing data was performed.

### 2.8. Analyses

Implant failure was the dichotomous outcome measure evaluated. The statistical unit for ‘implant failure’ was the implant. The untransformed proportion of failure for implants that perforated the maxillary sinus floor was calculated by considering the prevalence reported in the studies.

Pairwise meta-analysis was performed for the comparison of the failure rates bewteen implants placed in augmented sinuses in which the sinus membrane was perforated and implants placed in sinuses with no perforated membrane. Heterogeneity was checked using the I^2^ statistic. The inverse variance method was used for the random-effects (heterogeneity *p* < 0.10) or fixed-effects model (heterogeneity *p* ≥ 0.10) [20]. The estimates of relative effect for dichotomous outcomes were expressed in odds ratio (OR).

In order to explore the possible heterogeneity of effect between studies, a meta-regression was performed in order to verify how the OR was associated with the time of follow-up.

A funnel plot (plot of effect size versus standard error) was drawn.

The data were analyzed using the statistical software OpenMeta [Analyst], version 64-bit for Windows 10 [21]. The funnel plot was generated with the software OpenMEE, version 64-bit for Windows 10 [22].

## 3. Results

### 3.1. Literature Search

**Figure 1:**
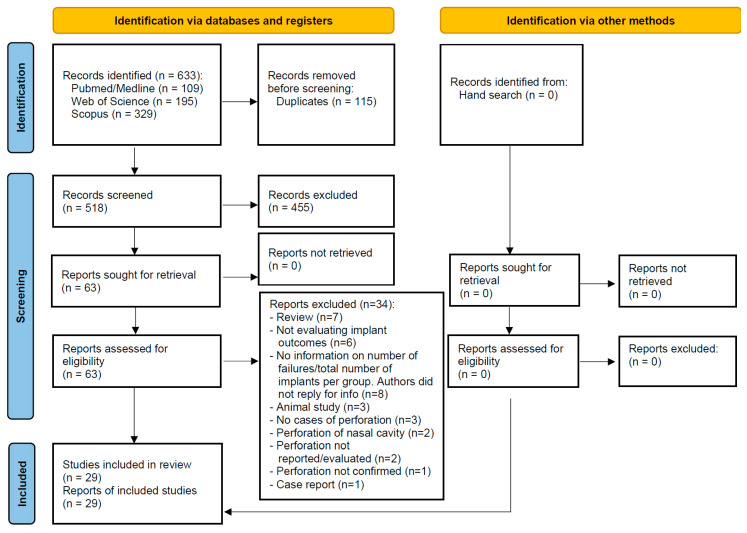
Study screening process.

The study selection process is summarized in Figure 1. The search strategy in the databases resulted in 633 papers (109 in PubMed/Medline, 195 in Web of Science, 329 in Scopus). A total of 115 articles were cited in more than one database (duplicates). The reviewers independently screened the titles and abstracts of the remaining entries, leading to the exclusion of 455 articles as the studies were not related to the subject. Of the full-text reports of the remaining 63 articles, there was a lack of information concerning either the number of failures or the total number of implants in cases with perforated sinus membrane in 8 studies. The authors of these 8 studies were contacted by e-mail up to three times asking for missing essential information for the analyses, but none of them replied. Other 26 studies did not fulfill the eligibility criteria. Hand-searching of journals and of the reference lists of selected studies did not yield additional papers. Thus, 29 studies were included in the review.

### 3.2. Description of the Studies

Out of the 29 included studies, 11 studies [23,24,25,26,27,28,29,30,31,32,33] reported on cases in which maxillary sinus perforation happened after installation of dental implants in the posterior maxilla, and 18 studies [34,35,36,37,38,39,40,41,42,43,44,45,46,47,48,49,50,51] reported on maxillary sinus membrane perforation during sinus lift procedure. Table S1 (see Supplementary Materials) presents detailed data of the 29 included studies.

#### 3.2.1. Maxillary Sinus Perforation after Installation of Dental Implants

The articles were published between 1984 and 2022. Two of the studies were multicenter, whereas the other nine studies were unicenter, one was prospective, ten were retrospective. Seven studies were conducted in a university, two in private practices, one study in a university and private practice, with no clear information about it for one study.

In six of the studies no implant failures were observed [23,24,28,29,30,33]. One of the studies [26] was cross-sectional and investigated the possible influence of penetrating implants on the status of maxillary sinuses on CBCT exams. The failure rates of perforating implants in the remaining studies were 28.9% [25], 1.6% [31], 4% [27], and 20% [32].

The development of transient sinusitis was reported in only one case [31]. The most common radiological sign reported, although not in every patient of every study, was mucosal thickening around the penetrating implants, limited to the maxillary sinus floor, with no pain nor discomfort.

#### 3.2.2. Maxillary Sinus Membrane Perforation during Sinus Lift Procedure

The articles were published between 2006 and 2023. One study was multicenter, whereas the other 17 studies were unicenter, 3 were prospective, 15 were retrospective. Twelve studies were conducted in a university, and six in private practices. All the studies for the exception of one [44] reported failure rates for the implants that were inserted in both the group of sinuses that presented and in the group of sinuses that did not present perforation of the sinus membrane during the sinus lifting procedure.

There were 1817 implants (73 failures) placed in augmented sinuses in which the sinus membrane was perforated and 5043 implants (274 failures) placed in sinuses with no perforated membrane. The results of the pairwise meta-analysis for this comparison are presented later on in this text.

Seven studies did not report/evaluate on the development of post-operative symptoms in the maxillary sinus [35,37,39,40,42,43,49]. For the studies that investigated symptoms, three studies reported absence of post-operative symptoms among the patients [34,50,51], while in the other eight studies [36,38,41,44,45,46,47,48] some cases of post-operative infection were observed, as well as some cases of post-operative sinusitis (see details in Table S1). Many of these patients were treated with antibiotics, with some sinuses being incised and drained.

### 3.3. Quality Assessment

All included studies were classified as “good” according to the quality assessment tool (see Supplementary Material). In most cases the main issues in the publications were related to not well-described statistical methods, and to the inclusion of non-consecutive patients in the studies.

### 3.4. Meta-Analysis

**Figure 2:**
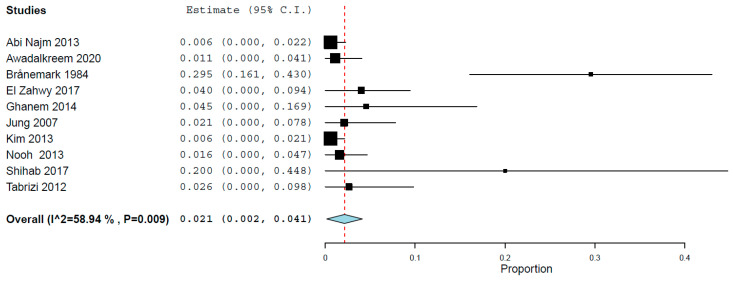
Estimated failure rate of implants that perforated the maxillary sinus floor [23,24,25,27,28,29,30,31,32,33]. Red dashed line: overall effect.

Ten studies reported the failure rate of implants when these perforated the floor of the maxillary sinus during placement. The estimated failure rate was of 2.1% (95% confidence interval 0.2%, 4.1%; standard error 1.0%, *p* = 0.035; heterogeneity: τ^2^ = 0.000, Chi^2^ = 21.917, I^2^ = 58.936, *p* = 0.009) (Figure 2). If the study of Brånemark et al. [25], with turned (machined) implants, is not included, the estimated failure rate goes down to 1.0% (95% confidence interval 0.0%, 1.9%; standard error 0.5%, *p* = 0.048; heterogeneity: τ^2^ = 0.000, Chi^2^ = 4.744, I^2^ = 0.000, *p* = 0.785).

**Figure 3:**
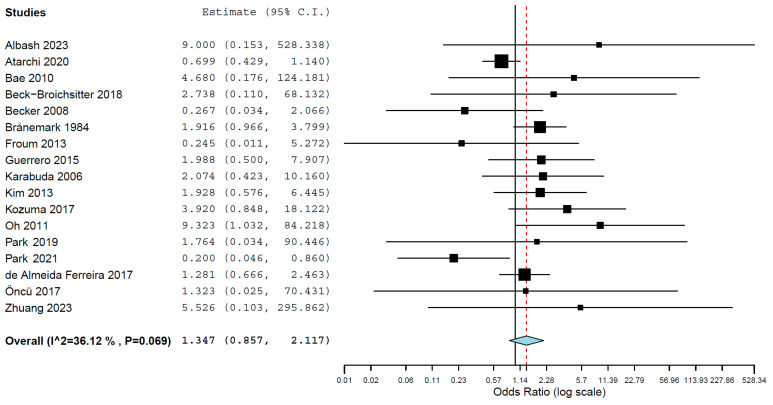
Forest plot for the event ‘implant failure’, for implants placed in the posterior maxilla, between procedures that presented and did not present sinus membrane perforation during max-illary sinus lift [25,34,35,36,37,38,39,40,41,43,45,46,47,48,49,50,51]. Red dashed line: overall effect.

Seventeen studies reported the results of implant failure for two groups, namely, patients that presented and did not present sinus membrane perforation during the sinus lift procedure. A random-effects model was used to evaluate the comparison of the implant failure between the groups, due to heterogeneity (τ^2^ = 0.241, Chi^2^ = 25.048, I^2^ = 36.121, *p* = 0.069). The pairwise meta-analysis showed implants placed in augmented sinuses in which the membrane was perforated did not have a higher risk of failure than implants placed in sinuses with no perforated membrane, with an OR 1.347 (95% confidence interval, 0.857, 2.117, *p* = 0.197; Figure 3). However, the effect size was not statistically significant.

### 3.5. Meta-Regression

Of the 17 studies that had two groups (patients that presented and did not present sinus membrane perforation), 16 provided clear information about the follow-up time or mean follow-up time. For the remaining one study, no information on precise follow-up time was available.

**Figure 4:**
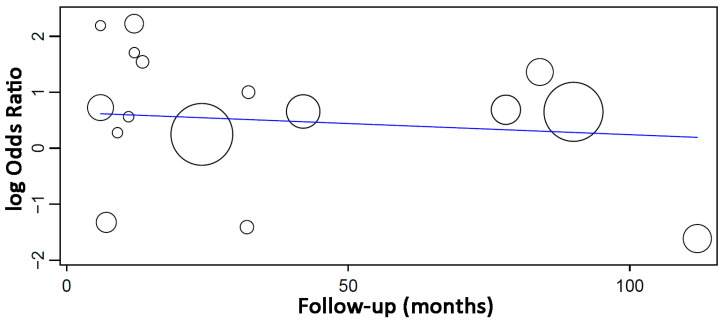
Scatter plot for the meta-regression with the association between the log odds ratio (OR) of implant failure between patients that presented sinus membrane perforation and patients that did not present sinus membrane perforation, and the follow-up time (in months). Every circle represents a study and the size of the circle represents the weight of the study in the analysis. The blue line represents the fitted line plot.

When a meta-regression considering the follow-up period as a covariate in relation to OR was plotted for these 16 studies, it was observed that the OR decreased with and increased follow-up time, although without significance (*p* = 0.500) (Figure 4). The first-degree equation resulted from the linear regression of this meta-regression was

y = 0.640 − 0.004x

where:

Intercept = 0.640 (−0.093, 1.374), standard error 0.374, *p* = 0.087
Follow-up = −0.004 (−0.016, 0.008), standard error 0.006, *p* = 0.500

### 3.6. Publication Bias

**Figure 5:**
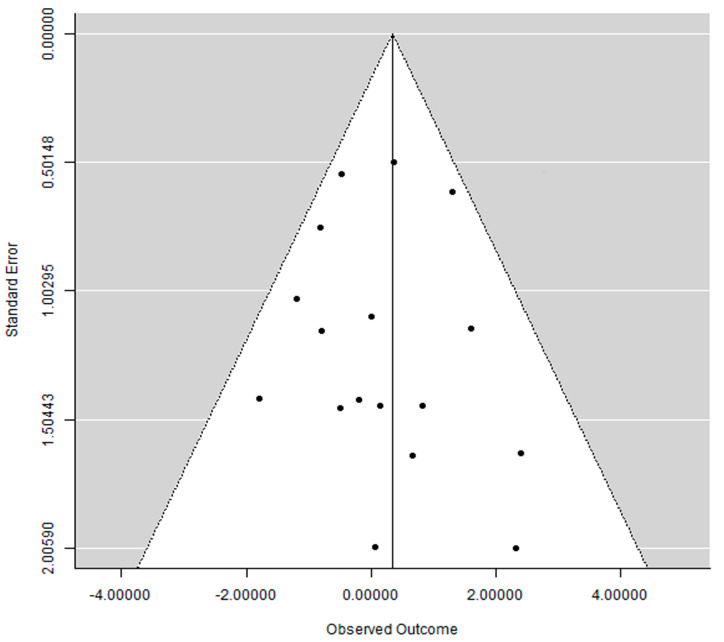
Funnel plot. Each dot represents a study. The area within the white triangle represents the 95% confidence interval.

The funnel plot did not show a clear asymmetry (Figure 5), indicating possible absence of publication bias.

## 4. Discussion

The purpose of the present review was to investigate the implant failure rates and complications after the perforation of the maxillary sinus by the placement of dental implants in posterior region of the maxilla, and after maxillary sinus membrane perforation during sinus lift procedure.

The results showed that the implant failure rate is generally low for implants penetrating in the floor of the maxillary sinus. Even though mucosal thickening around the penetrating implants was not an uncommon observation, only few cases developed symptoms in the sinus. The possible reason for the low implant failure rate and the low degree of symptomatology can be due to the fact that sinus perforation, in the great majority of the cases, may not result in any adverse occurrence. It was observed that formation of bone takes place around the apical part of implants intentionally protruded into the sinus floor without perforation of the sinus membrane, even without the addition of any grafting material [52]. Complications may not occur even when the membrane is perforated. It was observed in an animal study with dogs that implant perforation is not associated with sinus complications or pathologies, regardless of the extension of the implant protrusion into the sinus—protrusions of up to 8 mm into the sinus were investigated in this study [53]. This same research group later published the results of a retrospective clinical study in humans, in which 23 implants were intentionally intruded into the maxillary sinus of 9 patients, without sinus augmentation. After being followed up from 6 to 10 months, none of the patients presented signs of sinusitis, although mucous thickening of the sinus was a common observation [29]. A recent retrospective study based on computed tomography scans observed that the perforation of the maxillary sinus floor by implants is commonly associated sinus opacification, which occurrence is suggested to be more influenced by the implant diameter than by the length of the implant that is intruded into the sinus [54]. Mucosal thickening and opacification can occur, however, even when no perforation has taken place, being associated to when implants in the posterior maxilla are located within 2 mm from the cortex of the maxillary sinus floor [26].

The estimated failure of implants when these perforated the maxillary sinus floor was low, but it would become even lower if the early study of Brånemark et al. [25] would not be included in the analysis. The much higher failure rate for perforating implants observed in aforementioned study in comparison to the other studies can be associated with the use of turned (machined) implants in this study. Turned implants present a much higher risk of failure than the surface-modified implants [55,56] used in more recent studies included in the present review. Implants with moderately roughened surfaces usually present higher survival rates due to their chemical and topographical modified surface that favors osseointegration [56].

The results of the meta-analysis suggest that implants placed in augmented sinuses in which the sinus membrane was perforated do not present a higher risk of failure in comparison to implants placed in sinuses with no perforated membrane. Most surgeons perform some kind of treatment to address the perforated membrane, and with appropriate treatment, which differ depending on the size of the perforation [57], intraoperative sinus membrane perforations do not represent a higher risk for implant failure [38]. The implant failure rate, however, may vary significantly with the perforation size, with larger perforations resulting in higher failure rates, as observed in two studies [39,42].

Cases of augmented sinuses resulting in sinusitis or infection were reported, that needed to be treated with antibiotics, and some cases were even surgically drained. The occurrence of these post-operative complications could be associated with the occurrence of membrane perforation, as it was observed that grafted sinuses that had the membrane perforated during surgery presented a much higher incidence of sinusitis or infection in comparison to the cases in which a perforation did not occur [58]. The graft may dislodge into the sinus with membrane perforation, consequently potentially disrupting the normal sinus physiology [59,60]. It must be kept in mind that is not only during surgery that the sinus membrane can tear, but overfilling of the maxillary sinus with graft material may cause necrosis of the membrane and secondary perforation [61,62]. The perforation of the membrane may lead to the disrupted function of mucociliary apparatus, besides loss of the biologic barrier, resulting in an increase of sinus bacteria invasion which may evolve to infection [63,64]. Moreover, it seems that the occurrence of postoperative chronic sinusitis could be limited to patients with a predisposition for this condition due to deviation in form and size of the inferior turbinate and the position of the nasal septum, with structural drainage problems of the paranasal sinuses [62].

One must weigh the available options in face of the possible complications. The placement of short implants, which would be an option when one would like to avoid grafting procedures, provided that there is a minimum height of bone, has a higher risk of failure in comparison to longer implants [65], while sinus lift, which has a prevalence of membrane perforation going from 22 to 50% [57], may result in displacement of the graft material into the sinus leading to postoperative sinusitis and/or infection [63,64].

Limitations of the present review include the fact that there was a considerable number of confounding factors. There was no information about how many implants were inserted and failed in several different conditions for most (if not all) of the studies. Studies reported the presence of diabetics among the patients, as well as smokers, bruxers, and patients taking bisphosphonates. All these factors could have had a considerable impact on implant failure rates [66,67,68,69]. Moreover, the implants were placed by groups of different operators, due to different studies, which may also negatively influence implant survival rates, due to the variation in the surgeons’ technique, skills, and/or judgment [70]. Furthermore, the retrospective nature of many studies results in flaws manifested by the gaps in information. In addition, several studies presented small cohort sizes and short follow-ups.

## 5. Conclusions

Implant failure rate is generally low for implants penetrating in the floor of the maxillary sinus. Moreover, implants placed in augmented sinuses in which the sinus membrane was perforated do not present a higher risk of failure in comparison to implants placed in sinuses with no perforated membrane. The prevalence of postoperative infection or sinusitis is low, and it may depend either on the dimensions of the perforation or on the anatomical predisposition.

## Data Availability

All the data resulted from this review is presented in the manuscript.

## Supplementary Materials

The following supporting information can be downloaded at: https://www.mdpi.com/article/10.3390/jcm13051253/s1, List of journals included in the manual (hand) searching; List of excluded articles; Table S1. Detailed data of the included studies; Table S2. Quality assessment of the included studies, according to the National Institutes of Health (NIH); PRISMA 2020 Checklist.
