# Supplementary material for: Clinical Outcomes of Maxillary Sinus Floor Perforation by Dental Implants and Sinus Membrane Perforation during Sinus Augmentation: A Systematic Review and Meta-Analysis
Source: J Clin Med. 2024 Feb 22;13(5):1253. doi: 10.3390/jcm13051253 (PMC10932102; doi:10.3390/jcm13051253)
Supplement: Supplementary file 1 [file jcm-13-01253-s001.zip › jcm-2862424-supplementary.pdf]

SUPPLEMENTARY MATERIAL

Clinical outcomes of sinus membrane and maxillary sinus implant perforation: a systematic review and meta-analysis

a. **List of journals included in the manual (hand) searching**
b. **List of excluded articles**
c. **Table S1. Detailed data of the included studies**
d. **Table S2. Quality assessment of the included studies, according to the National Institutes of Health (NIH)**
e. **PRISMA 2020 Checklist**

a. List of journals included in the manual (hand) searching

Clinical Implant Dentistry and Related Research, Clinical Oral Implants Research, European Journal of Oral Implantology, Implant Dentistry, International Journal of Implant Dentistry, International Journal of Oral and Maxillofacial Implants, International Journal of Oral Implantology, International Journal of Prosthodontics, Journal of Clinical Periodontology, Journal of Oral Implantology, Journal of Periodontology, Journal of Prosthetic Dentistry, Journal of Prosthodontics, and Journal of Prosthodontic Research

b. List of excluded articles

1. Alhayati JZ, Al-Anee AM. Evaluation of crestal sinus floor elevations using versah burs with simultaneous implant placement, at residual bone height ≥ 2.0 _ < 6.0 mm. A prospective clinical study. Oral Maxillofac Surg. 2023 Jun;27(2):325-332.
2. Al-Juboori MJ. Progressive immediate loading of a perforated maxillary sinus dental implant: a case report. Clin Cosmet Investig Dent. 2015 Jan 29;7:25-31.
3. Chen TW, Chang HS, Leung KW, Lai YL, Kao SY. Implant placement immediately after the lateral approach of the trap door window procedure to create a maxillary sinus lift without bone grafting: a 2-year retrospective evaluation of 47 implants in 33 patients. J Oral Maxillofac Surg. 2007 Nov;65(11):2324-8.
4. Deng Y, Tong C, Gao K, Dou Y, Ma R, He Y, Li B, Liu W, Ma P. Modified internal sinus elevation for patients with low residual bone height: A retrospective clinical study. Clin Implant Dent Relat Res. 2023 Jun;25(3):458-472.
5. Díaz-Olivares LA, Cortés-Bretón Brinkmann J, Martínez-Rodríguez N, Martínez-González JM, López-Quiles J, Leco-Berrocal I, Meniz-García C. Management of Schneiderian membrane perforations during maxillary sinus floor augmentation with lateral approach in relation to subsequent implant survival rates: a systematic review and meta-analysis. Int J Implant Dent. 2021 Jul 12;7(1):91.
6. Elhamruni LM, Marzook HA, Ahmed WM, Abdul-Rahman M. Experimental study on penetration of dental implants into the maxillary sinus at different depths. Oral Maxillofac Surg. 2016 Sep;20(3):281-7.
7. Fermergård R, Astrand P. Osteotome sinus floor elevation and simultaneous placement of implants--a 1-year retrospective study with Astra Tech implants. Clin Implant Dent Relat Res. 2008 Mar;10(1):62-9.
8. Ferrigno N, Laureti M, Fanali S. Dental implants placement in conjunction with osteotome sinus floor elevation: a 12-year life-table analysis from a prospective study on 588 ITI implants. Clin Oral Implants Res. 2006 Apr;17(2):194-205.
9. Gao J, Yin W, Liu Y, Zhao X, Huangphattarakul V, Qu Y, Man Y. Cushioned grind-out technique transcrestal sinus floor elevation for simultaneous implantation in severe atrophic maxilla: A retrospective study with up to 7 years of follow-up. Clin Oral Implants Res. 2023 Jul;34(7):727-740.
10. Jung JH, Choi BH, Zhu SJ, Lee SH, Huh JY, You TM, Lee HJ, Li J. The effects of exposing dental implants to the maxillary sinus cavity on sinus complications. Oral Surg Oral Med Oral Pathol Oral Radiol Endod. 2006 Nov;102(5):602-5.

11. Kayabasoglu G, Nacar A, Altundag A, Cayonu M, Muhtarogullari M, Cingi C. A retrospective analysis of the relationship between rhinosinusitis and sinus lift dental implantation. Head Face Med. 2014 Dec 15;10:53.
12. Kim JS, Choi SM, Yoon JH, Lee EJ, Yoon J, Kwon SH, Yeo CD, Ryu JS, Lee JH, You YS, Kim SG, Lee MH, Han BH. What Affects Postoperative Sinusitis and Implant Failure after Dental Implant: A Meta-analysis. Otolaryngol Head Neck Surg. 2019 Jun;160(6):974-984.
13. Mardinger O, Nissan J, Chaushu G. Sinus floor augmentation with simultaneous implant placement in the severely atrophic maxilla: technical problems and complications. J Periodontol. 2007 Oct;78(10):1872-7.
14. Moraschini V, Uzeda MG, Sartoretto SC, Calasans-Maia MD. Maxillary sinus floor elevation with simultaneous implant placement without grafting materials: a systematic review and meta-analysis. Int J Oral Maxillofac Surg. 2017 May;46(5):636-647.
15. Nemati M, Khodaverdi N, Hosn Centenero SA, Tabrizi R. Which factors affect the risk of membrane perforation in lateral window maxillary sinus elevation? A prospective cohort study. J Craniomaxillofac Surg. 2023 Jul-Aug;51(7-8):427-432.
16. Nolan PJ, Freeman K, Kraut RA. Correlation between Schneiderian membrane perforation and sinus lift graft outcome: a retrospective evaluation of 359 augmented sinus. J Oral Maxillofac Surg. 2014 Jan;72(1):47-52.
17. Park WB, Kim YJ, Kang KL, Lim HC, Han JY. Long-term outcomes of the implants accidentally protruding into nasal cavity extended to posterior maxilla due to inferior meatus pneumatization. Clin Implant Dent Relat Res. 2020 Feb;22(1):105-111.
18. Phadnaik M, Tripathi RK, Nigam M, Solanki A, Karemore V. Management and simultaneous implant placement of maxillary sinus membrane perforation: A report of two cases. J Indian Soc Periodontol. 2020 Sep-Oct;24(5):477-480.
19. Pjetursson BE, Rast C, Brägger U, Schmidlin K, Zwahlen M, Lang NP. Maxillary sinus floor elevation using the (transalveolar) osteotome technique with or without grafting material. Part I: Implant survival and patients' perception. Clin Oral Implants Res. 2009 Jul;20(7):667-76.
20. Raghoebar GM, Batenburg RH, Timmenga NM, Vissink A, Reintsema H. Morbidity and complications of bone grafting of the floor of the maxillary sinus for the placement of endosseous implants. Mund Kiefer Gesichtschir. 1999 May;3 Suppl 1:S65-9.
21. Ragucci GM, Elnayef B, Suárez-López Del Amo F, Wang HL, Hernández-Alfaro F, Gargallo-Albiol J. Influence of exposing dental implants into the sinus cavity on survival and complications rate: a systematic review. Int J Implant Dent. 2019 Feb 5;5(1):6.
22. Schwarz L, Schiebel V, Hof M, Ulm C, Watzek G, Pommer B. Risk Factors of Membrane Perforation and Postoperative Complications in Sinus Floor Elevation Surgery: Review of 407 Augmentation Procedures. J Oral Maxillofac Surg. 2015 Jul;73(7):1275-82.
23. Seigneur M, Hascoët E, Chaux AG, Lesclous P, Hoornaert A, Cloitre A. Characteristics and management of dental implants displaced into the maxillary sinus: a systematic review. Int J Oral Maxillofac Surg. 2023 Feb;52(2):245-254.
24. Shao Q, Li J, Pu R, Feng Y, Jiang Z, Yang G. Risk factors for sinus membrane perforation during lateral window maxillary sinus floor elevation surgery: A retrospective study. Clin Implant Dent Relat Res. 2021 Dec;23(6):812-820.
25. Starch-Jensen T, Bruun NH, Spin-Neto R. Outcomes following osteotome-mediated sinus floor elevation with Bio-Oss Collagen or no grafting material: a one-year single-blind randomized controlled trial. Int J Oral Maxillofac Surg. 2023 Sep;52(9):988-997.
26. Testori T, Clauser T, Saibene AM, Artzi Z, Avila-Ortiz G, Chan HL, Chiapasco M, Craig JR, Felisati G, Friedland B, Gianni AB, Jensen OT, Lechien J, Lozada J, Misch CM, Nemcovsky C, Peacock Z, Pignataro L, Pikos MA, Pistilli R, Rasperini G, Scarfe W, Simion M, Stacchi C, Taschieri S, Trimarchi M, Urban I, Valentini P, Vinci R, Wallace SS, Zuffetti F, Del Fabbro M, Francetti L, Wang HL. Radiographic protrusion of dental implants in the maxillary sinus and nasal fossae: A multidisciplinary consensus utilising the modified Delphi method. Int J Oral Implantol (Berl). 2022 Sep 9;15(3):265-275.
27. Testori T, Weinstein T, Taschieri S, Wallace SS. Risk factors in lateral window sinus elevation surgery. Periodontol 2000. 2019 Oct;81(1):91-123.
28. Tidwell JK, Blijdorp PA, Stoelinga PJ, Brouns JB, Hinderks F. Composite grafting of the maxillary sinus for placement of endosteal implants. A preliminary report of 48 patients. Int J Oral Maxillofac Surg. 1992 Aug;21(4):204-9.

29. Timmenga NM, Raghoebar GM, Boering G, van Weissenbruch R. Maxillary sinus function after sinus lifts for the insertion of dental implants. J Oral Maxillofac Surg. 1997 Sep;55(9):936-9;discussion 940.
30. Uckan S, Deniz K, Dayangac E, Araz K, Ozdemir BH. Early implant survival in posterior maxilla with or without beta-tricalcium phosphate sinus floor graft. J Oral Maxillofac Surg. 2010 Jul;68(7):1642-5.
31. Uckan S, Tamer Y, Deniz K. Survival rates of implants inserted in the maxillary sinus area by internal or external approach. Implant Dent. 2011 Dec;20(6):476-9.
32. Wen Y, Wei D, Jiang X, Zhang Y, Di P, Lin Y. Lateral sinus floor elevation in patients with sinus floor defects: A retrospective study with a 1- to 9-year follow-up. Clin Oral Implants Res. 2023 Oct;34(10):1141-1150.
33. Yeom HG, Huh KH, Yi WJ, Heo MS, Lee SS, Choi SC, Kim JE. Nasal cavity perforation by implant fixtures: case series with emphasis on panoramic imaging of nasal cavity extending posteriorly. Head Face Med. 2023 Aug 22;19(1):37.
34. Zhong W, Chen B, Liang X, Ma G. Experimental study on penetration of dental implants into the maxillary sinus in different depths. J Appl Oral Sci. 2013 Nov-Dec;21(6):560-6.

c. <u>Table S1. Detailed data of the included studies.</u>

Study	Year	Study Design	Country / Setting	Perforating implants/ Patients (male/ female) (n)	Patients' age range (mean) (years)	Sinus augmentation	Smokers (n)	Implant outcomes<br>Sinus signs / symptoms
Albash	2023	RS (unicenter)	Syria / University	3/29 (18/11)	33-55 (46.2)	Transalveolar, with no graft	Light smokers	No postoperative complications
Atarchi	2020	RS (multicenter)	USA / University	-/1343 [b] (516/827)	18-96 (61.7)	Yes<br>No	58	616 implants placed in sinuses that had the membrane perforated, 21 implant failures<br>1707 implants in non-perforated sinuses, 82 implant failures<br>Signs/symptoms in sinus not reported/investigated
Bae	2010	PS (unicenter)	South Korea / University	-/16 [b] (9/7)	36-68 (52.3)	Yes	0	16 sinuses, of 6 with membrane perforation<br>13 implants placed in sinuses that had the membrane perforated, 1 implant failure<br>19 implants in non-perforated sinuses, no implant failures<br>Sinusitis developed in 2 patients, one of them had perforation. Treated with antibiotics + incision and drainage
Beck-Broichsitter	2018	RS (unicenter)	Germany / University	-/31 (12/19)	NA (61)	Yes	NA	92 implants (31 patients) placed in sinuses that had the membrane perforated, 1 implant failure<br>83 implants (31 patients) in non-perforated sinuses, no implant failure<br>Signs/symptoms in sinus not reported/investigated

Becker	2008	PS (unicenter)	Germany / University	-/201 [b] (84/117)	NA (55)	Yes	NA	93 implants (41 patients) placed in sinuses that had the membrane perforated, 1 implant failure<br>1 patient: sinusitis<br>332 implants (160 patients) in non-perforated sinuses, 13 implant failures<br>1 patient: sinusitis and local infection
de Almeida Ferreira	2017	RS (unicenter)	Brazil / Private practice	-/531 [b] (202/329)	18-82 (56)	Yes (graft)	NA	523 implants placed in sinuses that had the membrane perforated, 15 implant failures<br>1065 implants in non-perforated sinuses, 24 implant failures<br>Signs/symptoms in sinus not reported/investigated
Froum	2013	RS (unicenter)	USA / University	-/23 [b] (10/23)	46-75 (59)	Yes	Yes (no exact number)	35 implants placed in sinuses that had the membrane perforated, no implant failures<br>45 implants in non-perforated sinuses, 2 implant failures<br>Signs/symptoms in sinus not reported/investigated
Guerrero	2015	RS (unicenter)	Colombia / Private practice	-/68 (34/34)	32-78 (55)	Yes	NA	13 out of 101 sinuses perforated<br>16 implants placed in sinuses that had the membrane perforated, 3 implant failures<br>125 implants in non-perforated sinuses, 13 implant failures<br>3 patients: sinusitis (one had membrane perforation)
Hernández-Alfaro	2008	RS (unicenter)	Spain / Private practice	-/338 [b] (150/188)	27-69 (48)	Yes	NA	272 implants (85 patients) placed in sinuses that had the membrane perforated, 25 implant failures<br>894 implants in non-perforated sinuses (number of failures not reported for this group)<br>Signs/symptoms in sinus not reported/investigated
Karabuda	2006	RS (unicenter)	Turkey / University	-/91 (54/29)	29-74 (46)	Yes	No	26 implants placed in 12 sinuses that had the membrane perforated, 2 implant failures<br>233 implants in non-perforated sinuses, 9 implant failures<br>Signs/symptoms in sinus not reported/investigated
Kim	2013	RS (unicenter)	South Korea / University	-/30 (19/11)	18-78 (51.5)	Yes	NA	30 implants placed in sinuses that had the membrane perforated, 7 implant failures (4 patients)<br>44 implants in non-perforated sinuses, 6 implant failures (5 patients)<br>All these cases presented sinusitis (out of 338 sinuses in 259 patients)
Kim	2016	RS (unicenter)	South Korea / University	-/41 (28/13)	20-72 (57.2)	Yes	NA	99 implants placed in sinuses that had the membrane perforated, no implant failures<br>14 patients (34.1%): prescribed additional antibiotics due to postoperative infection or maxillary sinusitis<br>8 patients (19.5%): mild sinusitis - prescribed metronidazole together with amoxicillin/clavulanic acid 7 additional days

								6 patients (14.6%): Infection symptoms (e.g., pain, swelling, localized fever on operative region) without radiographic sign - prescribed amoxicillin/clavulanic acid 7 additional days<br>10 patients (24.4%): Nasal congestion or rhinorrhea - prescribed pseudoephedrine 7 days
Kozuma	2017	RS (unicenter)	Japan / University	-/109 [b] (47/74)	32-76 (58.3)	Yes	13	Implant loss in 3 out of 18 sinuses with membrane perforation, and in 5 out of 103 sinuses without perforation<br>8 cases of postoperative infection, all in sinuses without perforation
Oh	2011	RS (unicenter)	USA / Private practice	-/128 [b] (NA)	NA	Yes	NA	134 implants placed in 60 sinuses that had the membrane perforated, 4 implant failures, 3 sinuses infected<br>304 implants in 115 non-perforated sinuses, 1 implant failure, 1 sinus infected
Öncü	2017	PS (unicenter)	Turkey / University	-/16 [b] (10/6)	NA (55)	Yes	NA	15 implants placed in sinuses that had the membrane perforated, no implant failures<br>20 implants in non-perforated sinuses, no implant failures<br>No signs of infection in the maxillary sinus
Park	2019	RS (unicenter)	South Korea / Private practice	-/23 (19/4)	NA (58.9)	Yes	10	44 implants placed in sinuses that had the membrane perforated, no implant failures<br>78 implants in non-perforated sinuses, no implant failures<br>The following postoperative complications were observed with higher rates in the perforation group compared to the nonperforation group: nasal bleeding and facial swelling
Park	2021	RS (unicenter)	South Korea / Private practice	-/221 (NA)	NA (50.5)	Yes	NA	106 implants placed in sinuses that had the membrane perforated, 2 implant failures<br>273 implants in non-perforated sinuses, 24 implant failures<br>Symptoms in sinus not reported/investigated, only variation in the membrane thickening
Zhuang	2023	RS (unicenter)	China / University	-/61 (30/31)	NA (50)	Yes (transalveolar)	9	61 implants, no failures<br>Membrane perforation in 9 cases<br>No obvious postoperative symptoms such as redness, swelling, pyorrhoea, bleeding, or other symptoms were observed
Abi Najm	2013	RS (unicenter)	Switzerland / University	83/70 (30/40)	26-89 (66)	No	7	83 perforating implants, no failures<br>Estimated implant penetration was ≤3mm in all cases<br>No sinusitis<br>Radiological bone healing process<br>2 patients: biannual episodes of maxillary fullness and discomfort related to flu

Awadalkreem	2020	RS (unicenter)	Sudan / University	45/NA (NA)	NA (63.6)	No	NA	45 perforating implants, no failures<br>No previous history of sinusitis<br>No post-operative symptoms
Brånemark	1984	RS (unicenter)	Sweden / University	NA/44 (NA)	NA	No	NA	44 implants placed in sinuses that had the membrane perforated, 13 implant failures<br>529 implants in non-perforated sinuses, 95 implant failures<br>No radiological indications of untoward mucosal or bone reactions
Chaves	2022	RS (unicenter)	Brazil / University	71/199 [a] (88/111)	NA (59)	Yes (graft)<br>No	NA	66 (14.4% of the implants of the study): apical third inside sinus: 23 mucosal thickening, 1 antral pseudo cyst, 7 non-specific opacification<br>5 (1.1%): >2/3 inside sinus: 3 mucosal thickening, 2 non-specific opacification
El Zahwy	2017	RS (unicenter)	Egypt / NA	50/35 (22/13)	35-55 (49)	Yes (osteotome)	4	50 perforating implants, 2 failures<br>No apparent clinical or radiological signs of sinusitis at baseline<br>Penetration of 2-6 mm into sinus<br>1 patient: sensation of nasal fullness and obstruction persisting for two months after implant placement<br>2 patients: biannual episodes of maxillary fullness and discomfort related to flu<br>No sinusitis<br>Some of the cases showed mucosal thickening near and around the penetrated implants in the sinus cavity (did not involve the osteomeatal complex)
Ghanem	2014	RS (unicenter)	Egypt / University	10/10 (4/6)	31-54 (42.5)	No	NA	10 perforating implants, no failures<br>No complications in the sinuses, no sinusitis, healthy mucosal thickening around the intruded part of the implants
Jung	2007	RS (multicenter)	South Korea / Private practice	23/9 (5/4)	49-61 (55)	No	NA	23 perforating implants, no failures<br>Penetration 4-7 mm into sinus<br>None with previous history of sinusitis<br>Mucosal thickening around 14 implants (limited to the sinus floor)<br>No radiographic signs of pathologic bone reactions or abnormal loss of anchoring bone
Kim	2013	RS (unicenter)	South Korea / Private practice	/39 (26/13)	21-80 (60)	No	NA	87 perforating implants, no failures<br>3 patients: Nasal bleeding after implant surgery
Nooh	2013	PS (unicenter)	Saudi Arabia / University	63/56 (19/37)	NA (52)	No	0	63 perforating implants, 1 failure<br>1 patient: sinusitis (10 days after implant)
Shihab	2017	RS (unicenter)	Iraq / University	70/35 (16/19)	35-65 (NA)	No	NA	10 perforating implants, 2 implant failures<br>3 patients: epistaxis postoperatively, only on surgery day<br>No sinusitis developed

Tabrizi	2012	RS (multicenter)	Iran / University + Private practice	18/13 (NA)	NA	No	NA	18 perforating implants, no failures<br>Penetration 3-5 mm into sinus<br>None with previous history of sinusitis<br>2 patients: sinus membrane thickening<br>3 patients: increasing opacity peripheral to implants<br>No pain, no tenderness

NA – not available; PS – prospective study; RS – retrospective study

[a] Total number of patients of the study – not all of them had sinus perforation by an implant

[b] Total number of patients of the study – not all of them had sinus perforation during sinus lift procedure

d. Table S2. Quality assessment of the included studies, according to the National Institutes of Health (NIH).

**Study**	**Year**	**Was the study question or objective clearly stated?**	**Was the study population clearly and fully described, including a case definition?**	**Were the cases consecutive?**	**Were the subjects comparable?**	**Was the intervention clearly described?**	**Were the outcome measures clearly defined, valid, reliable, and implemented consistently across all study participants?**	**Was the length of follow-up adequate?** [a]	**Were the statistical methods well-described?**	**Were the results well-described?**	**Total (n/9)**
Abi Najm	2013	1	1	1	1	1	1	1	0	0	7/9
Albash	2023	1	1	1	1	1	1	1	0	1	8/9
Atarchi	2020	1	1	1	1	1	1	1	0	0	7/9
Awadalkreem	2020	1	1	1	1	1	1	1	0	0	7/9
Bae	2010	1	1	0	1	1	1	1	1	1	8/9
Beck-Broichsitter	2018	1	1	1	1	1	1	1	0	1	8/9
Becker	2008	1	1	1	1	1	1	1	0	1	8/9
Brånemark	1984	1	1	1	1	1	1	1	1	1	9/9
Chaves	2022	1	1	1	1	1	1	1	0	1	8/9
de Almeida Ferreira	2017	1	1	0	1	1	1	1	0	1	7/9
El Zahwy	2017	1	1	1	1	1	1	1	0	1	8/9
Froum	2013	1	1	1	1	1	1	1	0	0	7/9
Ghanem	2014	1	1	0	1	1	1	1	0	1	7/9

Guerrero	2015	1	1	0	1	1	1	1	0	1	7/9
Hernández-Alfaro	2008	1	1	0	1	1	1	1	0	1	7/9
Jung	2007	1	1	0	1	1	1	1	0	1	7/9
Karabuda	2006	1	1	1	1	1	1	1	0	1	8/9
Kim	2013	1	1	0	1	1	1	1	0	1	7/9
Kim	2013	1	1	1	1	1	1	1	0	1	8/9
Kim	2016	1	1	0	1	1	1	1	0	1	7/9
Kozuma	2017	1	1	0	1	1	1	1	0	1	7/9
Nooh	2013	1	1	1	1	1	1	1	1	1	9/9
Oh	2011	1	1	1	1	1	1	1	0	1	8/9
Öncü	2017	1	1	0	1	1	1	1	1	1	8/9
Park	2019	1	1	1	1	1	1	1	0	0	7/9
Park	2021	1	1	1	1	1	1	1	0	1	8/9
Shihab	2017	1	1	0	1	1	1	1	0	1	7/9
Tabrizi	2012	1	1	0	1	1	1	1	1	1	8/9
Zhuang	2023	1	1	0	1	1	1	1	1	1	8/9

[a] 6 months of follow-up was chosen to be of adequate length.

e. PRISMA 2020 Checklist

Section and Topic	Item #	Checklist item	Location where item is reported
**TITLE**			
Title	1	Identify the report as a systematic review.	Title
**ABSTRACT**			
Abstract	2	See the PRISMA 2020 for Abstracts checklist.	Abstract
**INTRODUCTION**			
Rationale	3	Describe the rationale for the review in the context of existing knowledge.	Introduction
Objectives	4	Provide an explicit statement of the objective(s) or question(s) the review addresses.	Introduction
**METHODS**			
Eligibility criteria	5	Specify the inclusion and exclusion criteria for the review and how studies were grouped for the syntheses.	Methods
Information sources	6	Specify all databases, registers, websites, organisations, reference lists and other sources searched or consulted to identify studies. Specify the date when each source was last searched or consulted.	Methods
Search strategy	7	Present the full search strategies for all databases, registers and websites, including any filters and limits used.	Methods
Selection process	8	Specify the methods used to decide whether a study met the inclusion criteria of the review, including how many reviewers screened each record and each report retrieved, whether they worked independently, and if applicable, details of automation tools used in the process.	Methods

Section and Topic	Item #	Checklist item	Location where item is reported
Data collection process	9	Specify the methods used to collect data from reports, including how many reviewers collected data from each report, whether they worked independently, any processes for obtaining or confirming data from study investigators, and if applicable, details of automation tools used in the process.	Methods
Data items	10a	List and define all outcomes for which data were sought. Specify whether all results that were compatible with each outcome domain in each study were sought (e.g. for all measures, time points, analyses), and if not, the methods used to decide which results to collect.	Methods
	10b	List and define all other variables for which data were sought (e.g. participant and intervention characteristics, funding sources). Describe any assumptions made about any missing or unclear information.	Methods
Study risk of bias assessment	11	Specify the methods used to assess risk of bias in the included studies, including details of the tool(s) used, how many reviewers assessed each study and whether they worked independently, and if applicable, details of automation tools used in the process.	Methods
Effect measures	12	Specify for each outcome the effect measure(s) (e.g. risk ratio, mean difference) used in the synthesis or presentation of results.	Methods
Synthesis methods	13a	Describe the processes used to decide which studies were eligible for each synthesis (e.g. tabulating the study intervention characteristics and comparing against the planned groups for each synthesis (item #5)).	Methods
	13b	Describe any methods required to prepare the data for presentation or synthesis, such as handling of missing summary statistics, or data conversions.	Methods
	13c	Describe any methods used to tabulate or visually display results of individual studies and syntheses.	Methods
	13d	Describe any methods used to synthesize results and provide a rationale for the choice(s). If meta-analysis was performed, describe the model(s), method(s) to identify the presence and extent of statistical heterogeneity, and software package(s) used.	Methods
	13e	Describe any methods used to explore possible causes of heterogeneity among study results (e.g. subgroup analysis, meta-regression).	Methods
	13f	Describe any sensitivity analyses conducted to assess robustness of the synthesized results.	Methods
Reporting bias assessment	14	Describe any methods used to assess risk of bias due to missing results in a synthesis (arising from reporting biases).	Methods
Certainty assessment	15	Describe any methods used to assess certainty (or confidence) in the body of evidence for an outcome.	Methods
**RESULTS**			
Study selection	16a	Describe the results of the search and selection process, from the number of records identified in the search to the number of studies included in the review, ideally using a flow diagram.	Results
	16b	Cite studies that might appear to meet the inclusion criteria, but which were excluded, and explain why they were excluded.	Results
Study characteristics	17	Cite each included study and present its characteristics.	Table S1
Risk of bias in studies	18	Present assessments of risk of bias for each included study.	Table S2
Results of individual studies	19	For all outcomes, present, for each study: (a) summary statistics for each group (where appropriate) and (b) an effect estimate and its precision (e.g. confidence/credible interval), ideally using structured tables or plots.	Results
Results of syntheses	20a	For each synthesis, briefly summarise the characteristics and risk of bias among contributing studies.	Results
	20b	Present results of all statistical syntheses conducted. If meta-analysis was done, present for each the summary estimate and its precision (e.g. confidence/credible interval) and measures of statistical heterogeneity. If comparing groups, describe the direction of the effect.	Results
	20c	Present results of all investigations of possible causes of heterogeneity among study results.	Results

Section and Topic	Item #	Checklist item	Location where item is reported
	20d	Present results of all sensitivity analyses conducted to assess the robustness of the synthesized results.	Results
Reporting biases	21	Present assessments of risk of bias due to missing results (arising from reporting biases) for each synthesis assessed.	Results
Certainty of evidence	22	Present assessments of certainty (or confidence) in the body of evidence for each outcome assessed.	Results
**DISCUSSION**			
Discussion	23a	Provide a general interpretation of the results in the context of other evidence.	Discussion
	23b	Discuss any limitations of the evidence included in the review.	Discussion
	23c	Discuss any limitations of the review processes used.	Discussion
	23d	Discuss implications of the results for practice, policy, and future research.	Discussion
**OTHER INFORMATION**			
Registration and protocol	24a	Provide registration information for the review, including register name and registration number, or state that the review was not registered.	Methods
	24b	Indicate where the review protocol can be accessed, or state that a protocol was not prepared.	Methods
	24c	Describe and explain any amendments to information provided at registration or in the protocol.	Methods
Support	25	Describe sources of financial or non-financial support for the review, and the role of the funders or sponsors in the review.	End of manuscript
Competing interests	26	Declare any competing interests of review authors.	End of manuscript
Availability of data, code and other materials	27	Report which of the following are publicly available and where they can be found: template data collection forms; data extracted from included studies; data used for all analyses; analytic code; any other materials used in the review.	End of manuscript
